# Supplementary material for: Super-resolution proximity labeling with enhanced direct identification of biotinylation sites
Source: Commun Biol. 2024 May 9;7:554. doi: 10.1038/s42003-024-06112-w (PMC11082246; doi:10.1038/s42003-024-06112-w)
Supplement: Supplementary file 2 — Supplementary information [file 42003_2024_6112_MOESM2_ESM.pdf]

Supplementary information

Sanghee Shin[1,2,3,4†], Song-Yi Lee[5,6†], Myeong-Gyun Kang[5], Dong-Gi Jang[1,2], Jeesoo Kim[1,2], Hyun-Woo Rhee[2,5,*], and Jong-Seo Kim[1,2,*]

[1]Center for RNA Research, Institute of Basic Science, Seoul National University, Seoul 08826, Korea

[2]School of Biological Sciences, Seoul National University, Seoul 08826, Korea

[3]The Research Institute of Basic Science, Seoul National University, Seoul 08826, Korea

[4]Current address: Department of Cancer Biology, Dana-Farber Cancer Institute, Boston, MA, USA

[5]Department of Chemistry, Seoul National University, Seoul 08826, Korea

[6]Current address: Department of Genetics, Stanford University, Stanford, CA, USA.

[†] These authors contributed equally to this work.

*Correspondence to: jongseokim@snu.ac.kr and rheehw@snu.ac.kr

Supplementary Table 1. Construct Information

Name (expected size)	Features	Promotor/ Vector	Details
Mito-V5-APEX2 (unprocessed: 32 kDa, processed: 29 kDa)	*KpnI*-Mito-*BamHI*-V5-APEX2-Stop-*NotI*	CMV/ pcDNA5	Mito-: MLATRVFSLVGKRAISTSVCVRAH (matrix targeting sequence, Fornuskova et al., 2010)
ScoI-V5-APEX2 (unprocessed: 62 kDa, processed: 59 kDa)	*HindIII*-ScoI-*BamHI*-V5-APEX2-Stop-*XhoI*	CMV/ pcDNA5	Sco1 (NM_004589)
V5-APEX2-NES* (29.8 kDa)	*NotI*-V5-APEX2-NES-Stop-*XhoI*	CMV/ pcDNA3	NES: LQLPPLERLTLD (nuclear exclusion signal)*

The nuclear export sequence, NES (LQLPPLERLTLD), was derived from residues 6–17 of the HIV-1 Rev protein30. Protein processed size during translocation was obtained by programs: Mitoprot[1] (http://ihg.gsf.de/ihg/mitoprot.html or Mitoprot software is available by: ftp://ftp.biologie.ens.fr/pub/molbio) and SignalP 4.1 Server[2] (http://www.cbs.dtu.dk/services/SignalP/) .

*The imaging pattern of these constructs was previously characterized in Lee et al, Cell Rep. 2016, 15, 1837-1847.

Figure S1. Enrichment protocol comparison(commercial bead)

a

	Total PSM	Labeled PSM	Labeled unique-sequence	Corresponding labeled protein	Enrichment Efficiency (%)
GE_NA1	4522	3692	1387	509	81.65%
GE_NA2	4970	3848	1461	518	77.42%
GE_SA1	5363	4214	1416	509	78.58%
GE_SA2	5717	4474	1486	522	78.26%
Pierce_SA1	4317	3646	1292	476	84.46%
Pierce_SA2	5724	4479	1484	522	78.25%

Labeled protein FDR< 1%
Cut-off score >= 76.889

b

Biotinylation site

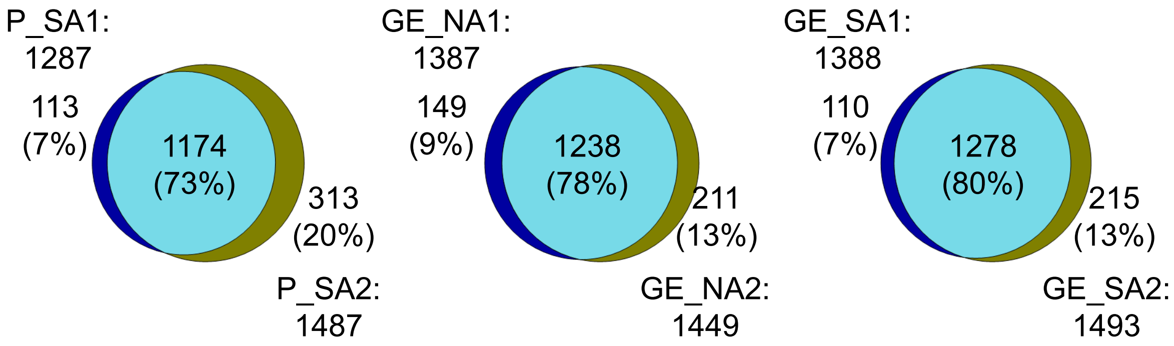

c

Protein Group
(>2 overlap)

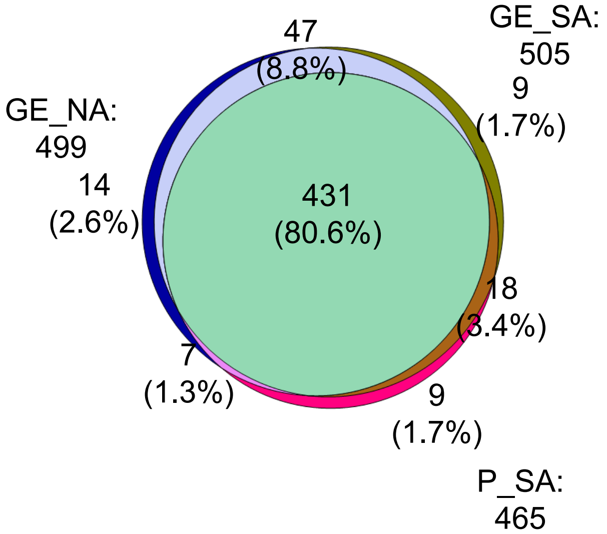

Figure S1. (a) Identification result of biotinylation sites using different avidin-based beads for enriching biotinylated peptides. Overlap analysis between replicates (b) and among different types of beads (c).

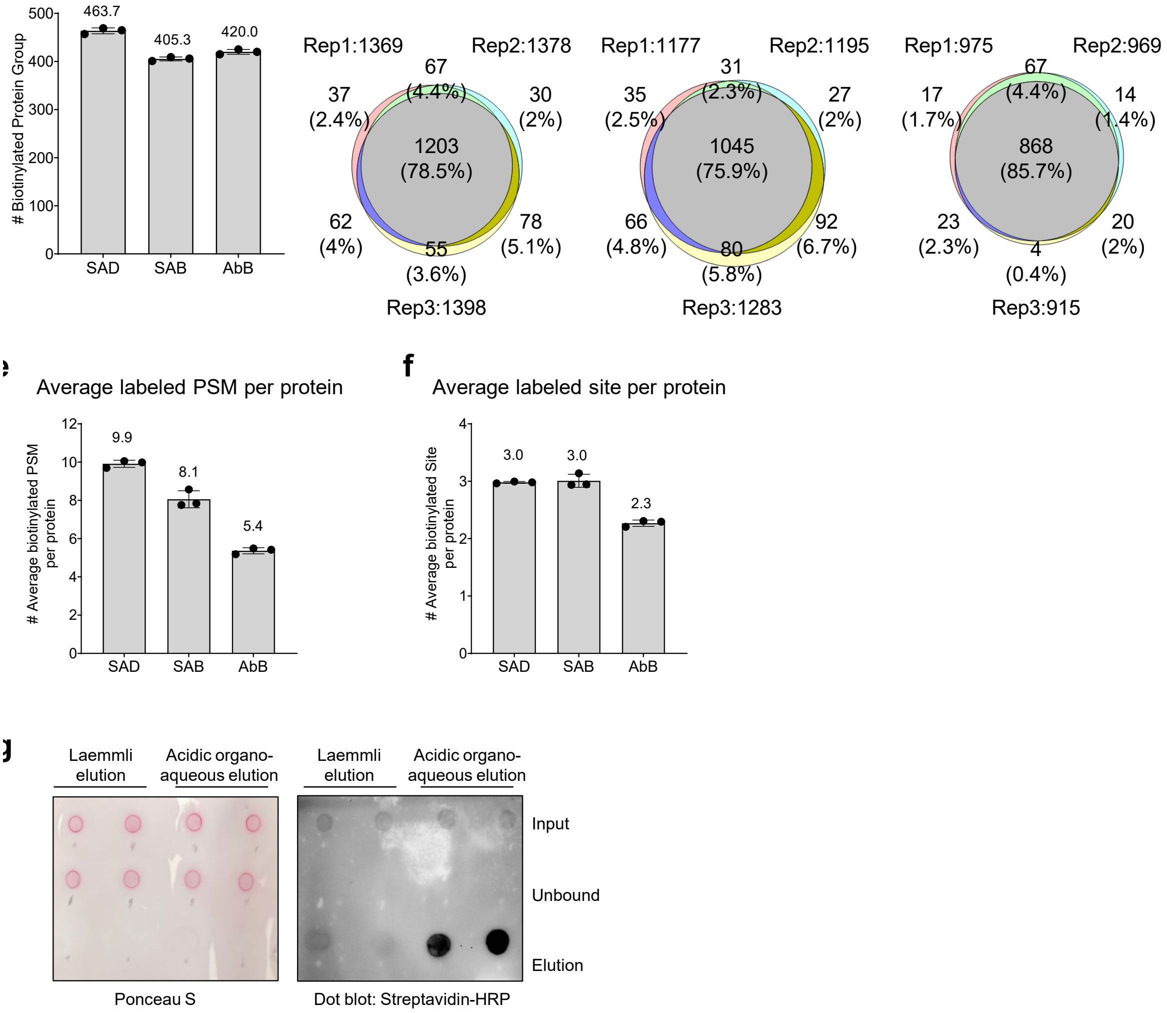

Figure S2. Performance comparison in terms of identified biotin labeled protein group(a) between various combinations of biotinylation site identification protocols and labeling probes. (b-d) Identification overlap between replicates among different protocols and probe combinations. Analysis result of average biotinylated PSM(e) and site(f) per protein identified among different approaches. (g) dot blot result of comparing different elution buffers

Figure S3. protocol comparison

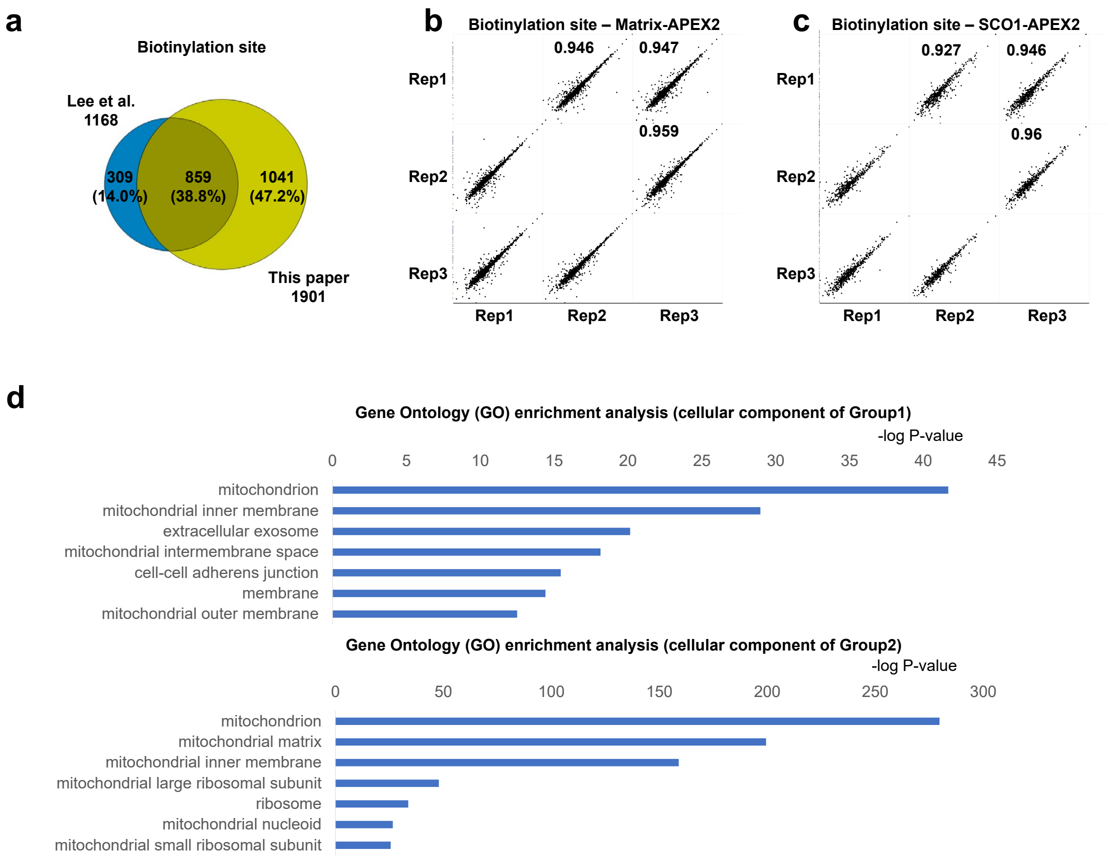

e

				Corresponding unique-peptide count		Corresponding unique-protein count	
#	Description	Lee et al, 2017 (SPOT-ID)	This study	Lee et al, 2017 (SPOT-ID)	This study	Lee et al, 2017 (SPOT-ID)	This study
1	# of Labeled sites detected	1168	1902	1635	2334	495	752
2	# of labeled site that map to proteins with Uniprot CC mitochondrial annotation	1012	1522	1437	1906	399	511
3	# of labeled site that map to transmembrane prot eins (predicted or known)	221	320	331	385	107	133
4	# of labeled site from 3 that map to proteins with mitochondrial annotation (Cellular component)	192	272	288	332	87	104
5	# of labeled site from 4 that map to proteins of kn own topology	78	99	126	131	33	36
6	# of labeled site from 5 that are correctly matche d to known topology	76	92	124	123	31	31

Figure S3f

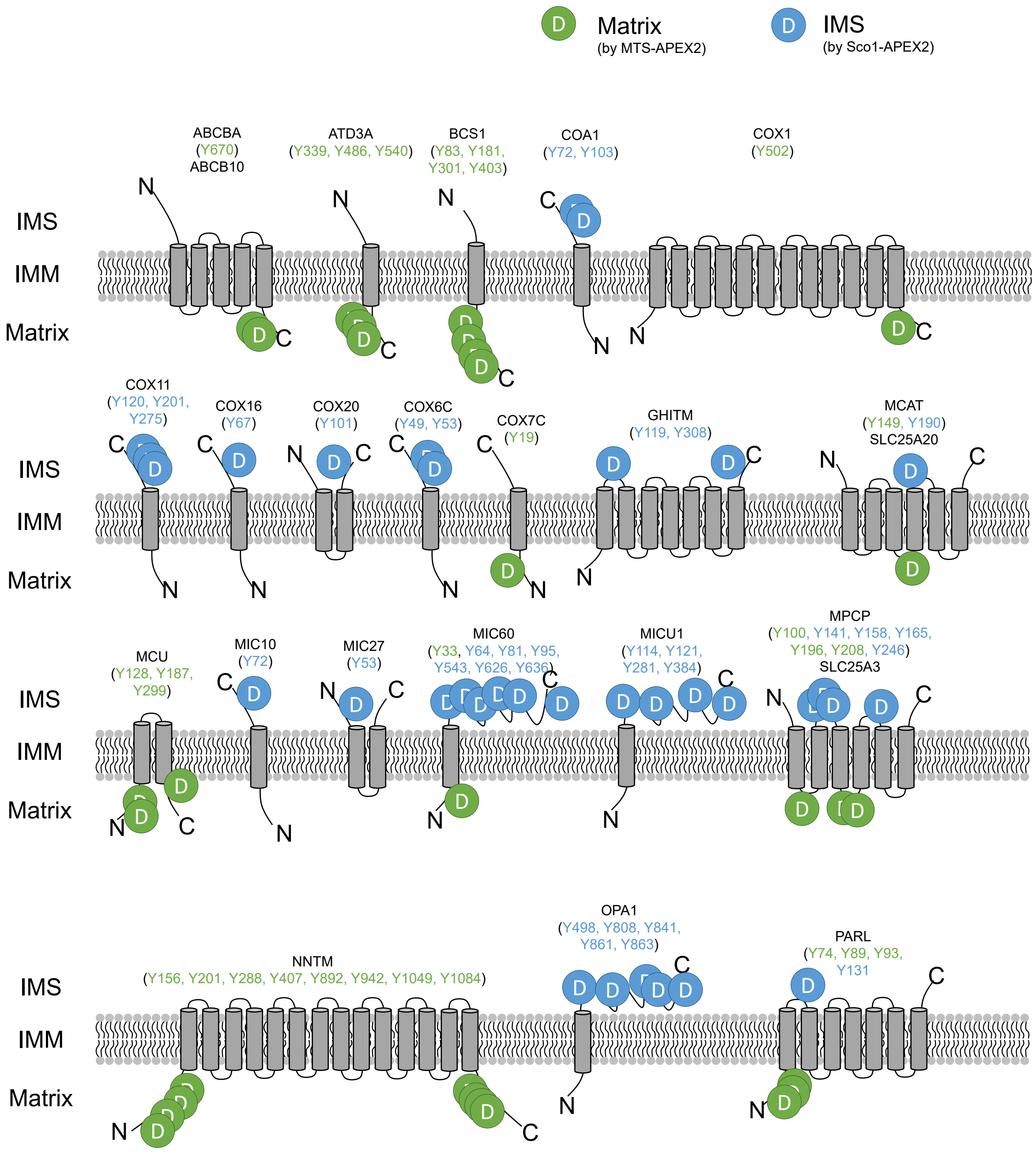

Matrix (by MTS-APEX2)
IMS (by Sco1-APEX2)
ABCBA (Y670) ABCB10
ATD3A (Y339, Y486, Y540)
BCS1 (Y83, Y181, Y301, Y403)
COA1 (Y72, Y103)
COX1 (Y502)
COX11 (Y120, Y201, Y275)
COX16 (Y67)
COX20 (Y101)
COX6C (Y49, Y53)
COX7C (Y19)
GHITM (Y119, Y308)
MCAT (Y149, Y190) SLC25A20
MCU (Y128, Y187, Y299)
MIC10 (Y72)
MIC27 (Y53)
MIC60 (Y33, Y64, Y81, Y95, Y543, Y626, Y636)
MICU1 (Y114, Y121, Y281, Y384)
MPCP (Y100, Y141, Y158, Y165, Y196, Y208, Y246) SLC25A3
NNTM (Y156, Y201, Y288, Y407, Y892, Y942, Y1049, Y1084)
OPA1 (Y498, Y808, Y841, Y861, Y863)
PARL (Y74, Y89, Y93, Y131)
IMS
IMM
Matrix

Figure S3g

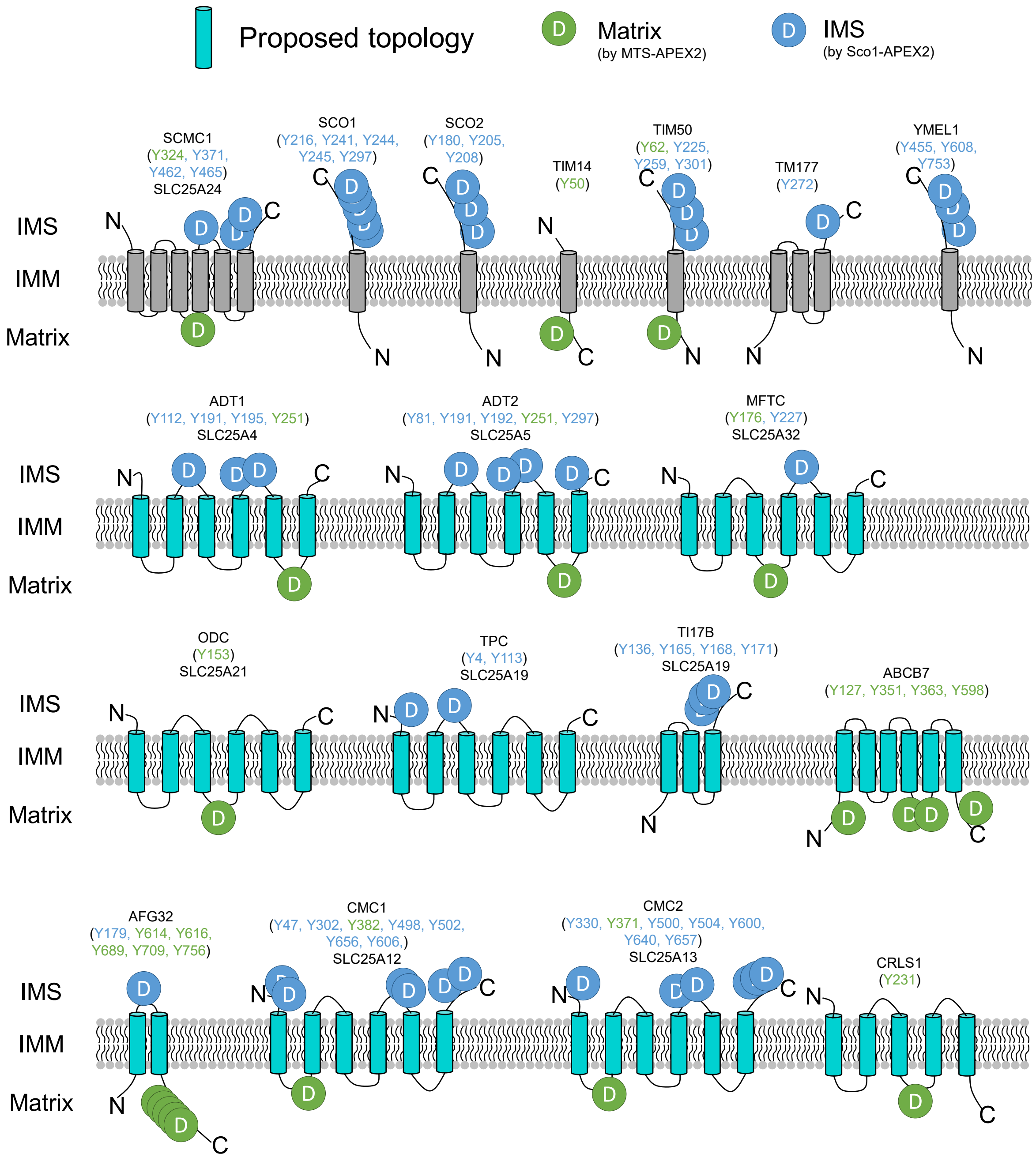
Proposed topology
Matrix
(by MTS-APEX2)
IMS
(by Sco1-APEX2)
SCMC1
(Y324, Y371, Y462, Y465)
SLC25A24
SCO1
(Y216, Y241, Y244, Y245, Y297)
SCO2
(Y180, Y205, Y208)
TIM14
(Y50)
TIM50
(Y62, Y225, Y259, Y301)
TM177
(Y272)
YMEL1
(Y455, Y608, Y753)
ADT1
(Y112, Y191, Y195, Y251)
SLC25A4
ADT2
(Y81, Y191, Y192, Y251, Y297)
SLC25A5
MFTC
(Y176, Y227)
SLC25A32
ODC
(Y153)
SLC25A21
TPC
(Y4, Y113)
SLC25A19
TI17B
(Y136, Y165, Y168, Y171)
SLC25A19
ABCB7
(Y127, Y351, Y363, Y598)
AFG32
(Y179, Y614, Y616, Y689, Y709, Y756)
CMC1
(Y47, Y302, Y382, Y498, Y502, Y656, Y606,)
SLC25A12
CMC2
(Y330, Y371, Y500, Y504, Y600, Y640, Y657)
SLC25A13
CRLS1
(Y231)
IMS
IMM
Matrix

Figure S3h

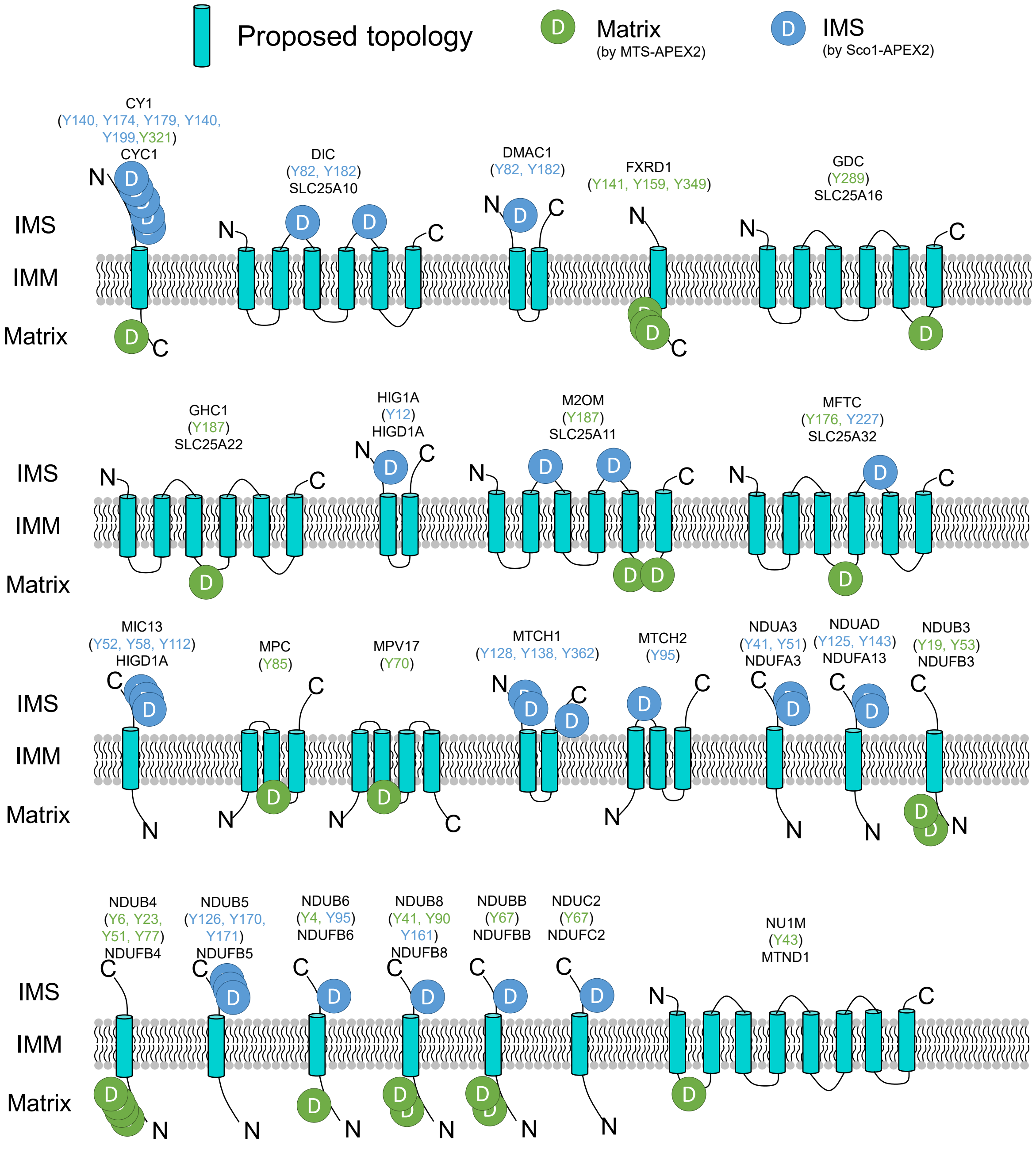

Proposed topology
D Matrix (by MTS-APEX2)
D IMS (by Sco1-APEX2)
IMS
IMM
Matrix
CY1 (Y140, Y174, Y179, Y140, Y199, Y321) CYC1
DIC (Y82, Y182) SLC25A10
DMAC1 (Y82, Y182)
FXRD1 (Y141, Y159, Y349)
GDC (Y289) SLC25A16
GHC1 (Y187) SLC25A22
HIG1A (Y12) HIGD1A
M2OM (Y187) SLC25A11
MFTC (Y176, Y227) SLC25A32
MIC13 (Y52, Y58, Y112) HIGD1A
MPC (Y85)
MPV17 (Y70)
MTCH1 (Y128, Y138, Y362)
MTCH2 (Y95)
NDUA3 (Y41, Y51) NDUFA3
NDUAD (Y125, Y143) NDUFA13
NDUB3 (Y19, Y53) NDUFB3
NDUB4 (Y6, Y23, Y51, Y77) NDUFB4
NDUB5 (Y126, Y170, Y171) NDUFB5
NDUB6 (Y4, Y95) NDUFB6
NDUB8 (Y41, Y90 Y161) NDUFB8
NDUBB (Y67) NDUFBB
NDUC2 (Y67) NDUFC2
NU1M (Y43) MTND1

Figure S3i

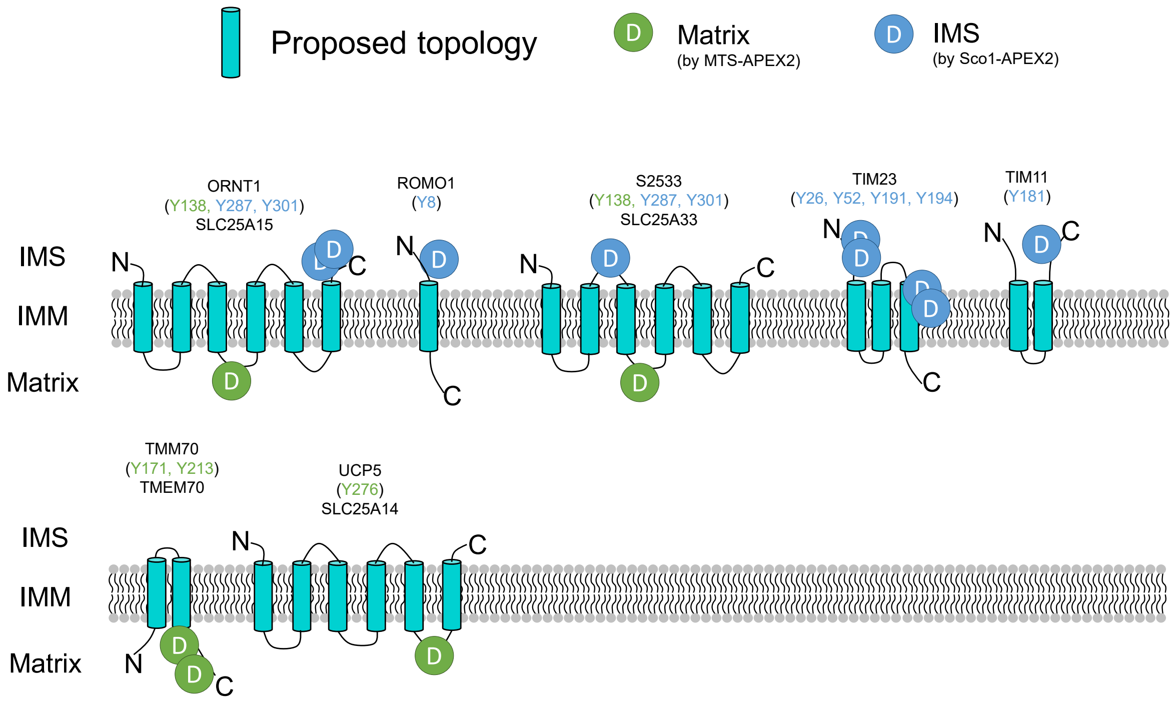

Figure S3. (a) Identification results of biotinylation site by SPOT-ID(Lee et al.) and newly developed method on biotinylated protein shown in figure 1e. Scatter plot analysis of reproducibility among identified biotinylation sites between replicates of MTS-apex2(b) and SCO1-APEX2(c) experiments based on LFQ intensity. (d) Gene ontology (GO) enrichment analysis results of protein groups that are shown in figure 1g. (e) Confirmed and proposed topology of transmembrane proteins located in the mitochondrial inner membrane (IMM).(f-i) application of identified of biotinylation sites on topology mapping
