# Supplementary material for: Super-resolution proximity labeling with enhanced direct identification of biotinylation sites
Source: Commun Biol. 2024 May 9;7:554. doi: 10.1038/s42003-024-06112-w (PMC11082246; doi:10.1038/s42003-024-06112-w)
Supplement: Supplementary file 3 — Description of Additional Supplementary Files [file 42003_2024_6112_MOESM3_ESM.pdf]

**Description of Additional Supplementary Files**

**File name:** Supplementary Data 1
**Description:** Identified list of biotinylated peptides, sites and proteins by newly developed site identification method applied to Matrix-APEX2 experiment.

**File name:** Supplementary Data 2
**Description:** Identified list of enriched proteins by conventional on-bead digestion method applied to Matrix-APEX2 experiment.

**File name:** Supplementary Data 3
**Description:** Identification summary of biotinylated peptide enrichment protocol comparison shown in figure S2.

**File name:** Supplementary Data 4
**Description:** Identified list of biotinylated sites of mitochondrial matrix and IMS related to figure 1e-i.

**File name:** Supplementary Data 5
**Description:** Enriched processing-body proteome via high-resolution mapping of proximity labled sites. Related to Figure 6.

**File name:** Supplementary Data 6
**Description:** Identified result of biotinylation sites using different avidin-based beads for enriching biotinylated peptides.
